# The interplay of TARG1 and PARG protects against genomic instability

**DOI:** 10.1016/j.celrep.2023.113113

**Published:** 2023-09-06

**Authors:** Joséphine Groslambert, Evgeniia Prokhorova, Anne R. Wondisford, Callum Tromans-Coia, Celeste Giansanti, Jennifer Jansen, Gyula Timinszky, Matthias Dobbelstein, Dragana Ahel, Roderick J. O’Sullivan, Ivan Ahel

**Affiliations:** 1 Sir William Dunn School of Pathology, University of Oxford, Oxford OX1 3RE, UK; 2 Department of Pharmacology and Chemical Biology, UPMC Hillman Cancer, University of Pittsburgh, Pittsburgh, PA, USA; 3 Department of Molecular Oncology, Göttingen Center of Molecular Biosciences (GZMB), University Medical Center Göttingen, Justus-von-Liebig-Weg 11, 37077 Göttingen, Germany; 4 Laboratory of DNA Damage and Nuclear Dynamics, Institute of Genetics, Biological Research Centre, Eötvös Loránd Research Network (ELKH), 6276 Szeged, Hungary

**Keywords:** DNA damage, ADP-ribosylation, PARP inhibitor, TARG1, PARG inhibitor, cancer

## Abstract

The timely removal of ADP-ribosylation is crucial for efficient DNA repair. However, much remains to be discovered about ADP-ribosylhydrolases. Here, we characterize the physiological role of TARG1, an ADP-ribosylhydrolase that removes aspartate/glutamate-linked ADP-ribosylation. We reveal its function in the DNA damage response and show that the loss of TARG1 sensitizes cells to inhibitors of topoisomerase II, ATR, and PARP. Furthermore, we find a PARP1-mediated synthetic lethal interaction between TARG1 and PARG, driven by the toxic accumulation of ADP-ribosylation, that induces replication stress and genomic instability. Finally, we show that histone PARylation factor 1 (HPF1) deficiency exacerbates the toxicity and genomic instability induced by excessive ADP-ribosylation, suggesting a close crosstalk between components of the serine- and aspartate/glutamate-linked ADP-ribosylation pathways. Altogether, our data identify TARG1 as a potential biomarker for the response of cancer cells to PARP and PARG inhibition and establish that the interplay of TARG1 and PARG protects cells against genomic instability.

## Highlights

• TARG1 deficiency leads to DNA repair defects that can be exploited therapeutically
• TARG1 and PARG suppression is synthetically lethal and induces replication stress
• TARG1 levels could predict cancer cell sensitivity to PARP and PARG inhibition
• TARG1 and PARG protect cells against toxic PARP1-mediated ADP-ribosylation

## Graphical abstract

**Figure:**
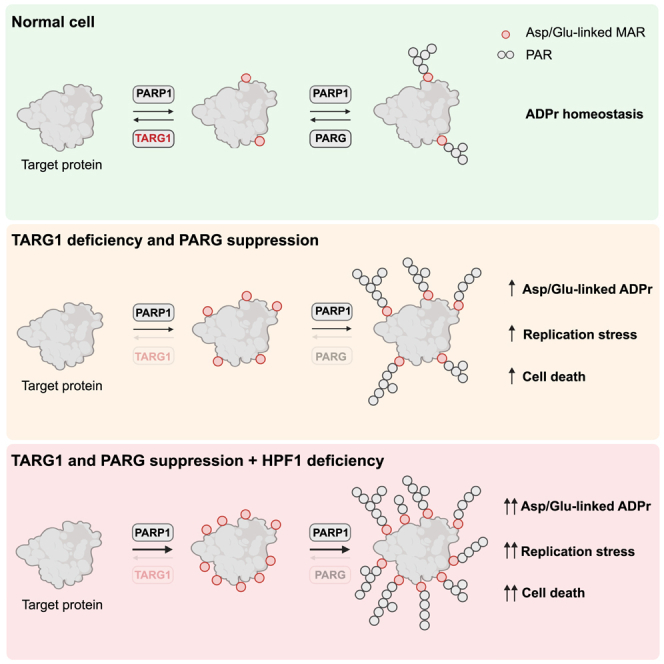

## Teaser

Groslambert et al. identify TARG1 and PARG as factors protecting cells from toxic PARP1-mediated aspartate/glutamate-linked ADP-ribosylation and genotoxic stress. This underpins the finding of synthetic lethality between TARG1 and PARG, which is further exacerbated by HPF1 deficiency and reveals vulnerabilities in cancer cells.

## Introduction

ADP-ribosylation (ADPr) is a post-translational modification (PTM) with a role in many cellular processes, including DNA damage repair, chromatin remodeling, and RNA metabolism.^1^ ADP-ribosyltransferases (ARTs) catalyze the modification by transferring an ADP-ribose unit from NAD^+^ onto target proteins with the release of nicotinamide.^2^^,^^3^ Poly(ADP-ribose) polymerases (PARPs), the best-characterized ART family, have been widely researched for their role in the DNA damage response (DDR), with the best-studied member, PARP1, thought to account for about 85% of cellular ADPr upon DNA damage.^4^^,^^5^ PARP1 swiftly binds to DNA breaks and attaches mono- and poly-ADP-ribose (MAR and PAR, respectively) on many protein targets, including itself, DNA repair proteins, histones, and chromatin-remodeling factors.^6^^,^^7^^,^^8^

PARylation of target proteins promotes many downstream events, such as the recruitment of DNA repair machineries accompanied by chromatin decondensation, which facilitates access of repair factors to sites of DNA damage.^6^^,^^9^^,^^10^ In response to DNA damage, ADPr is most robustly initiated on serine residues and is performed by PARP1 or PARP2 (PARP1/2) forming a complex with histone PARylation factor 1 (HPF1).^11^^,^^12^^,^^13^^,^^14^ In addition, PARP1/2 are capable of initiating ADPr on aspartate/glutamate residues, the regulation of which remains poorly understood.^15^^,^^16^^,^^17^^,^^18^

Timely reversal of ADPr is key to prevent the trapping of proteins recruited to DNA damage sites and for promoting access to downstream repair factors.^19^^,^^20^ PAR glycohydrolase (PARG) is the major hydrolase of ADPr in the cell and cleaves the ribose–ribose bonds between PAR subunits but is unable to cleave the bond that links the first MAR moiety to the target protein.^21^^,^^22^^,^^23^ ADP-ribosylhydrolase 3 (ARH3/ADPRHL2) is the hydrolase that specifically reverses serine-linked MAR.^24^^,^^25^ Persistent serine-linked PARylation upon ARH3 deficiency and PARG suppression has been shown to be highly cytotoxic, leading to dysregulation of histone acetylation, transcription, and telomere elongation.^25^

The synthesis of aspartate/glutamate-linked MAR does not require HPF1 and is reversed *in vitro* by terminal ADP-ribose protein glycohydrolase (TARG1/OARD1/C6orf13), MacroD1, and MacroD2.^26^^,^^27^^,^^28^ The exact cellular function and physiological targets of TARG1 remain to be elucidated, but initial investigations of the cellular function of TARG1 suggested a role in the DDR, with TARG1 being recruited to the sites of laser-induced DNA damage in a PARP1/2-dependent manner.^28^ Moreover, a homozygous mutation in the *TARG1* gene has been reported in patients with severe neurodegenerative disease.^28^ This suggests that, similarly to PARG and ARH3, TARG1 plays an essential role in protecting cells from the toxic accumulation of ADPr. Despite the importance of PARP1/2 inhibitors (PARPis) in cancer treatment and the growing interest in PARG as a therapeutic target,^29^^,^^30^^,^^31^^,^^32^ the relative contribution of TARG1 and PARG in the modulation of ADPr levels and cellular homeostasis remains to be elucidated.

Here, we characterize the role of TARG1 in DNA damage repair by showing that TARG1 loss sensitizes cells to topoisomerase II, ATR, and PARPi. Moreover, we reveal a synthetic lethality relationship between TARG1 and PARG driven by the toxic accumulation of ADPr. Our data demonstrate that TARG1 contributes to the reversal of endogenous cellular ADPr and to the prevention of excessive replication stress.

## Results

### The loss of TARG1 sensitizes cells to topoisomerase II and ATR inhibition and induces homologous recombination defects

**Figure 1:**
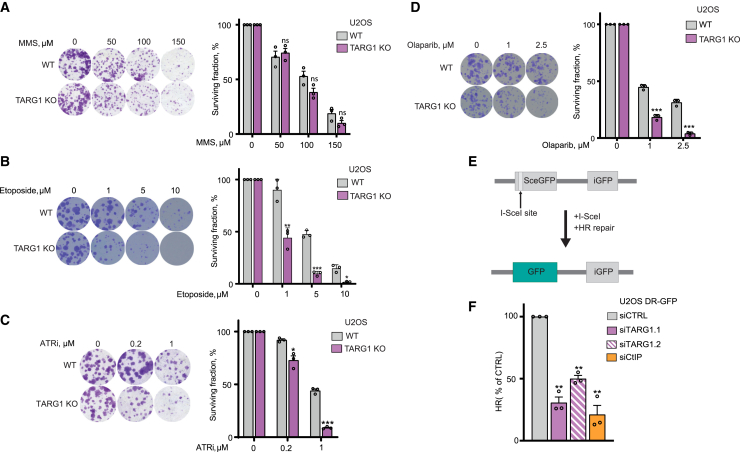
The loss of TARG1 sensitizes cells to topoisomerase II and ATR inhibition and induces homologous recombination defects (A–D) Representative images (left) and quantification (right) of colony-formation assays with U2OS WT and TARG1-KO cells treated with DMSO or as indicated. (E) Schematic representation of the DR-GFP HR reporter assay, as described.^33^ The *SceGFP* gene is a GFP gene mutated to contain a recognition site for the I-*Sce* I endonuclease and two in-frame stop codons. The *iGFP* gene is a truncated internal WT *GFP* fragment. Following *I-Sce I* expression, *SceGFP* is cleaved, yielding a DSB. A functional GFP gene is restored upon repair via HR using iGFP as a donor sequence. (F) U2OS DR-GFP cells were transfected with non-targeting siRNA control (siCTRL), two different siTARG1, or siCtIP and 24 h later were cotransfected with I-SceI and mCherry for 48 h prior to analysis by flow cytometry. The proportion of GFP-positive cells among the mCherry-positive population was used as a readout for I-SceI-induced HR events. CtIP knockdown acts as a positive control here.^34^ Data are shown as mean ± SD, n = 3 (A–D), or mean ± SEM, n = 3 (F); ns, not significant, ^∗^p < 0.05, ^∗∗^p < 0.01, and ^∗∗∗^p < 0.001 (two-tailed Student’s t test). See also Figure S1.

To clarify the role of TARG1 in DNA repair, we used the U2OS cell line as a well-established model in the DNA repair field and sought to test the sensitivity of U2OS TARG1-KO (knockout) cells to several DNA-damaging agents by performing long-term colony-formation assays. U2OS TARG1-KO cells (Figure S1A) displayed no significantly increased sensitivity to MMS (Figure 1A) or to H_2_O_2_ (Figure S1B), two standard ADPr-inducing genotoxic agents that induce high levels of serine-linked ADPr.^35^^,^^36^^,^^37^ By investigating the sensitivity of U2OS TARG1-KO cells to a broader range of DNA-damaging agents, we found that TARG1 loss sensitized U2OS cells to a topoisomerase II inhibitor, etoposide (Figure 1B), which acts by trapping topoisomerase II onto DNA, thereby creating double-stranded breaks (DSBs) and perturbing DNA replication.^38^^,^^39^ We next assessed sensitivity to the inhibition of the replication stress response kinase ATR using the ATR inhibitor (ATRi) VE-821.^40^ We observed that U2OS TARG1-KO cells displayed increased sensitivity to ATR inhibition (Figure 1C). Genetic complementation with wild-type (WT) TARG1, but not catalytically inactive K84A TARG1, rescued the sensitivity of TARG1-KO cells to ATR inhibition (Figure S1C),^28^ confirming that ATRi sensitivity was driven by the loss of TARG1 ADP-ribosylhydrolase activity.

Given the potential role of TARG1 in reversing PARP1/2-dependent ADPr and that PARPis have been shown to induce replication stress and perturb DNA replication,^41^ we also assessed the response of TARG1-KO cells to the PARPi olaparib. U2OS cells showed marked sensitivity to olaparib upon TARG1 loss (Figure 1D), contrary to the PARPi resistance that was previously observed upon deficiency of the serine-specific ADP-ribosylhydrolase ARH3.^25^^,^^42^ This result highlights the complexity of ADPr signaling and suggests that unlike elevated serine-linked ADPr levels,^42^ elevated aspartate/glutamate-linked ADPr levels do not protect from but rather sensitize cells to PARP1/2 inhibition.

**Figure 2:**
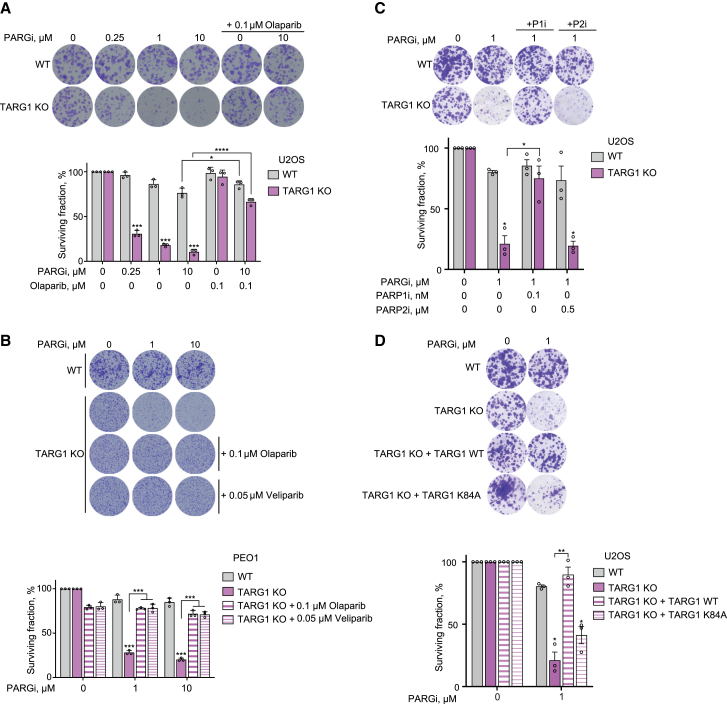
TARG1 deficiency is synthetically lethal with PARG suppression in a PARP1-dependent manner (A–D) Representative images (top) and quantification (bottom) of colony-formation assays with U2OS WT and TARG1-KO cells (A, C, and D), PEO1 WT and TARG1-KO cells (B), and U2OS TARG1-KO cells complemented with TARG1 WT or catalytically inactive K84A mutant (D) treated with DMSO or as indicated. (C) P1i, PARP1 inhibitor; P2i, PARP2 inhibitor. Data are shown as mean ± SD, n = 3; ^∗^p < 0.05, ^∗∗^p < 0.01, ^∗∗∗^p < 0.001, and ^∗∗∗∗^p < 0.0001 (two-tailed Student’s t test). See also Figure S2.

Homologous recombination (HR) deficiency is the best-characterized mechanism underlying sensitivity to PARPis.^43^^,^^44^^,^^45^ Thus, upon observing the sensitivity of U2OS TARG1-KO to PARPis, we sought to assess the effect of TARG1 loss on HR by knocking down TARG1 in U2OS cells carrying the HR reporter construct DR-GFP (Figures 2B and S2A).^33^ Following DSB induction by I-SceI, we observed a significant reduction in HR efficiency upon TARG1 knockdown, as measured by the proportion of GFP-positive cells (Figures 1F and S1E). Knockdown of the HR factor CtIP, serving as a positive control, led to a strong reduction of HR efficiency. These results further support the DNA damage repair function of TARG1 and indicate that the sensitivity of TARG1-KO cells to PARPis is at least partly due to the disruption of HR upon TARG1 loss.

### TARG1 deficiency is synthetically lethal with PARG suppression in a PARP1-dependent manner

Next, we sought to analyze the response of TARG1-KO cells to the PARG inhibitor (PARGi) PDD00017273,^46^ which blocks the reversal of PARylation. We showed that the loss of TARG1 led to a marked sensitization of U2OS cells to PARGi (Figures 2A and S2A), demonstrating a synthetic lethality relationship between TARG1 and PARG. Notably, this synthetic lethality occurs in the absence of exogenous DNA damage, suggesting that endogenous stimuli lead to high PAR levels and cell death. Given that PARG suppression was indicated as one of the mechanisms of PARPi resistance in BRCA2-deficient cancer cells,^47^ we additionally explored the effect of TARG1 loss on PARGi sensitivity in BRCA2-deficient ovarian cancer PEO1 cells and observed a similar sensitivity phenotype (Figures 2B and S2B).

In both TARG1-KO U2OS (Figures 2A and S2A) and PEO1 cells (Figures 2B, S2B, and S2C), the addition of low concentrations of a PARPi, olaparib or veliparib, rescued sensitivity to PARGi, indicating that the PARGi-induced toxicity is dependent on the enzymatic activities of PARP1 and/or PARP2. To differentiate between a PARP1- or PARP2-dependent phenotype, we attempted to rescue sensitivity to PARGi with AZD5305^48^ and UPF 1069,^49^ specific inhibitors for PARP1 and PARP2, respectively. The sensitivity of U2OS TARG1-KO cells to PARGi was rescued upon addition of a low concentration of the specific PARP1 inhibitor but not the PARP2 inhibitor (Figure 2C), indicating that this toxicity is caused by a PARP1-dependent ADPr overproduction. Complementation with WT, but not K84A, TARG1 rescued the sensitivity of U2OS TARG1-KO cells to PARG inhibition (Figure 2D), confirming that the sensitivity of TARG1-KO cells to PARGi was due specifically to the loss of TARG1 catalytic activity. Altogether, these results uncover a synthetically lethal relationship between TARG1 and PARG that is dependent on PARP1 activity.

### The joint loss of TARG1 and PARG activity leads to excessive ADPr and induces replication stress

**Figure 3:**
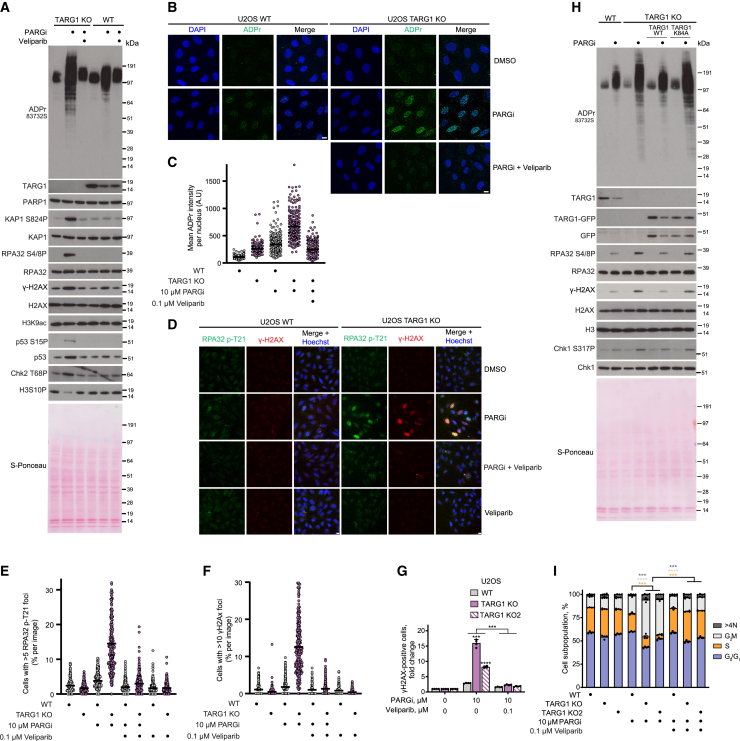
The joint loss of TARG1 and PARG activity leads to excessive ADPr and induces replication stress (A) U2OS cells were treated with DMSO, 10 μM PARGi and 0.1 μM veliparib, and 10 μM PARGi for 6 days. ADPr and DNA damage marker levels were analyzed using western blotting. (B) Representative images of ADPr staining in detergent pre-extracted cells treated with DMSO, 10 μM PARGi, or 10 μM PARGi and 0.1 μM veliparib for 4 days. Scale bars, 10 μm. A representative image from n = 3 is shown. (C) Quantification of (B). Each point represents the mean ADPr intensity of an individual nucleus. The black line represents the mean ADPr intensity of each condition; at least 220 cells were analyzed per condition. (D) Representative images of RPA32 p-T21 and γH2AX staining in cells treated with DMSO, 10 μM PARGi, 10 μM PARGi and 0.1 μM veliparib, or 0.1 μM veliparib for 4 days. Scale bars, 10 μm. A representative image from n = 3 is shown. (E and F) Quantification of (D). Each point represents the percentage of cells with >5 RPA32 p-T21 foci per image (E) or the percentage of cells with >10 γH2AX foci per image (F). The black line represents the mean percentage of cells per image with >5 RPA32 p-T21 (E) or >10 γH2AX (F) foci for each condition. ∼250 images and a total of ∼20,000 cells were analyzed per condition. (G) Quantification of γH2AX-positive cells by flow cytometry after 5 days of exposure to DMSO or indicated treatment. (H) U2OS cells were treated with DMSO or 10 μM PARGi for 4 days. ADPr and DNA damage marker levels were analyzed using western blotting. (I) Quantification of cell-cycle analysis by flow cytometry of EdU- and DAPI-stained U2OS cells after 5 days of exposure to DMSO or indicated treatment and 1 h EdU pulse. Data are shown as mean ± SEM of four independent experiments (G and I). ^∗∗∗^p < 0.001 and ^∗∗∗∗^p < 0.0001 (two-tailed Student’s t test). See also Figure S3.

We then wanted to confirm that this revealed synthetic lethality relationship between TARG1 and PARG was indeed caused by an excessive accumulation of ADPr. Prolonged treatment with PARGi led to a strong increase in ADPr levels in U2OS TARG1-KO cells, as observed by western blotting (Figure 3A). Detergent pre-extraction of cells prior to fixation, followed by immunofluorescence analysis, also showed a striking increase in ADPr in TARG1-KO cells upon PARGi treatment (Figures 3B and 3C). Conversely, PARGi treatment of U2OS WT cells led to a weaker enrichment of ADPr levels in comparison to PARGi-treated TARG1-KO cells (Figures 3A–3C). The addition of a low concentration of veliparib rescued the PARGi-induced signal in TARG1-KO cells (Figures 3A–3C), showing that this signal is PARP1/2 dependent. These results demonstrate that the synthetic lethality relationship observed between TARG1 and PARG is due to the accumulation of unreversed PARP1-mediated ADPr.

We then wanted to further characterize the molecular consequences of unregulated ADPr accumulation upon PARG and TARG1 deficiency. PARGi treatment of U2OS cells led to a subtle increase in replication protein A subunit 32 (RPA32) loading in WT cells and to a considerably more pronounced increase in TARG1-KO cells, indicating that TARG1-KO cells were challenged with higher levels of replication stress (Figures S3A and S3B). This was further supported by a strong induction in RPA32 phosphorylation of residue T21 (RPA32 p-T21) in U2OS TARG1-KO cells upon PARGi treatment. Indeed, TARG1 deficiency in U2OS cells caused a ∼4-fold increase in the percentage of PARGi-induced RPA32 p-T21 foci-positive cells (Figures 3D and 3E).

Sustained replication stress can lead to fork breakage and can be detected by increased levels of the DSB marker yH2AX.^50^ While PARGi treatment of WT cells led to a subtle increase in γH2AX-positive cells, the effect of PARGi treatment on γH2AX induction in U2OS TARG1-KO cells was far more striking, as we observed an ∼8-fold increase in the percentage of PARGi-induced γH2AX-foci-positive cells upon TARG1 loss (Figures 3D and 3F). Interestingly, we also noticed that the joint loss of TARG1 and PARG induced a significant increase in cells exhibiting a pan-nuclear high-intensity γH2AX signal, indicative of a global cellular DNA damage phenotype comprising hundreds of DNA breaks (Figures 3D and S3C). Elevated γH2AX levels were confirmed by western blotting (Figure 3A), and flow cytometry analysis showed an increased fraction of PARGi-induced γH2AX-positive cells in U2OS TARG1-KO cells (Figures 3G and S3E), with significant enrichment of γH2AX specifically in post-replicative EdU-positive cells (Figure S3D). DSBs can activate the ataxia telangiectasia mutated checkpoint kinase 2 (ATM/CHK2) signaling pathway.^50^^,^^51^ Consistently, PARGi treatment of U2OS TARG1-KO cells induced phosphorylation of ATM targets, including RPA32 (pS4/8), KAP1 (pS824), and CHK2 (pT68) (Figures 3A and 3H). Additionally, we observed activation of the ataxia telangiectasia and Rad3-related (ATR) checkpoint kinase 1 (ATR/CHK1) pathway, which orchestrates the replication stress response,^52^ through the phosphorylation of ATR targets such as CHK1 (pS317) and p53 (pS15) (Figures 3A and 3H). Furthermore, the induction of replication stress and genomic instability upon the joint loss of TARG1 and PARG was also observed in PEO1 cells, as indicated by the upregulation of KAP1 (pS824), RPA32 (pS4/8), and γH2AX (Figure S2C).

Importantly, both the induction of replication stress and DSB markers observed upon the joint loss of TARG1 and PARG activity were rescued with the addition of a low concentration of the PARPi veliparib (Figures 3A–3G and S3A–S3D), confirming that the unreversed accumulation of PARP1-dependent ADPr is driving this DNA damage phenotype. Genetic complementation of U2OS TARG1-KO cells with TARG1 WT, but not TARG1 K84A, restored the reversal of PARGi-induced ADPr and rescued the DNA damage phenotype (Figure 3H).

Lastly, we sought to determine whether the excessive accumulation of ADPr observed in TARG1-KO cells treated with PARGi would disrupt cell-cycle progression. PARGi treatment of TARG1-KO cells significantly decreased cell proliferation, as shown by reduced EdU incorporation, induced G_2_/M arrest, and a slight but significant increase in the percentage of polyploid cells with >4 N DNA content in TARG1-KO cells treated with PARGi (Figure 3I). The induction of G_2_/M arrest is consistent with the observed activation of the ATR/CHK1 pathway, which can be caused by the accumulation of replication-associated DNA lesions.^53^

Together, these results indicate that PARGi treatment of U2OS TARG1-KO cells led to an increase in RPA loading as well as to high levels of DSBs, thereby activating the ATR and ATM pathways. Furthermore, our results also show that the moderate increase of ADPr caused by the loss of PARG activity alone does not induce this DNA damage phenotype but that it is the excessive accumulation of PARP1-mediated ADPr detected upon the joint loss of TARG1 and PARG that leads to the sustained induction of DNA damage. Of note, the unregulated accumulation of aspartate/glutamate-linked ADPr did not disrupt acetylation of H3 on K9 (Figure 3A), a marker of active transcription regions,^54^ unlike what was observed for the unrestrained accumulation of serine-linked ADPr.^25^

### TARG1 and HPF1 both protect cells from toxic PARP1-mediated ADPr

**Figure 4:**
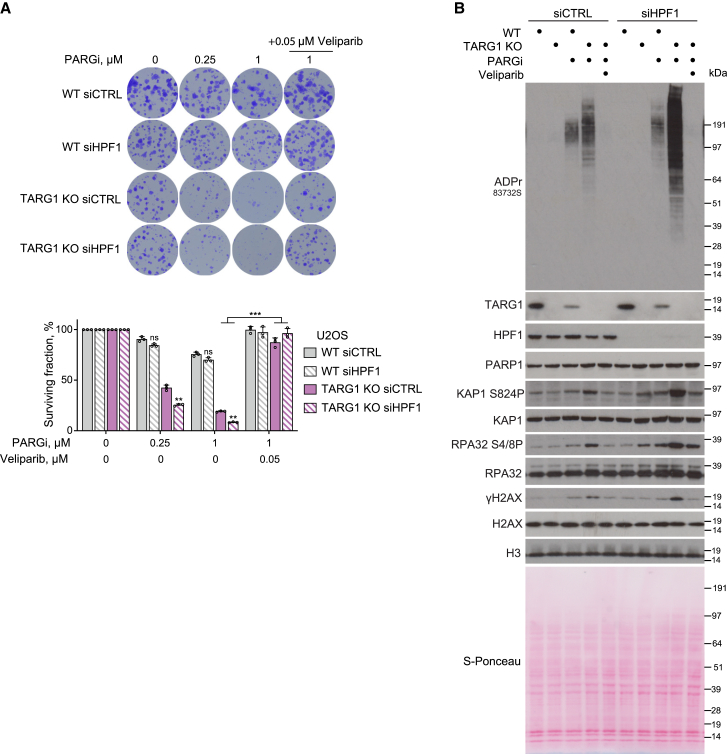
TARG1 and HPF1 both protect cells from toxic PARP1-mediated ADPr (A) Quantification of colony-formation assay with U2OS WT and TARG1-KO cells transfected with siCTRL or siHPF1 and treated with DMSO or as indicated. Data are shown as mean ± SD, n = 3; ^∗∗^p < 0.01 and ^∗∗∗^p < 0.001 (two-tailed Student’s t test). (B) U2OS cells transfected with siCTRL or siHPF1 were treated with DMSO, 10 μM PARGi, or 10 μM PARGi and 0.1 μM veliparib for 4 days. ADPr and DNA damage marker levels were analyzed using western blotting.

After showing the protective roles of TARG1 and PARG in restricting the toxic accumulation of ADPr and genomic instabilities, we sought to investigate the role of other factors known to constrain ADPr. We thus examined the role of HPF1, whose binding to PARP1 has been shown to restrict PAR chain length and steer the PARP1 activity towards serine sites,^12^^,^^13^^,^^55^ in protecting cells from toxic PARP1-mediated ADPr. HPF1 knockdown further sensitized U2OS TARG1-KO to PARGi treatment (Figure 4A). On the other hand, HPF1 knockdown in WT cells had no significant effect on PARGi sensitivity (Figure 4A). Furthermore, we report that HPF1 knockdown further increases the ADPr accumulation observed upon PARGi treatment of TARG1-KO cells (Figure 4B). Consistently, this increased ADPr accumulation is accompanied by an exacerbation of the genomic instability phenotype as shown by an increase in γH2AX, RPA2 (pS4/8), and KAP1 (pS824) levels (Figure 4B). The addition of a low concentration of veliparib completely reversed the PARGi-induced ADPr accumulation in TARG1-KO cells with knockdown of HPF1 as well as the increase in levels of DSB markers (Figure 4B), indicating that the excess ADPr observed upon HPF1 deficiency is also catalyzed by PARP1. Consistently, the addition of veliparib rescued sensitivity to PARGi of TARG1-KO cells with HPF1 knockdown (Figure 4A). Our results thus identify TARG1 and HPF1 as two key factors in the restraint of toxic non-serine-linked ADPr and in protection against genomic instability.

## Discussion

It has been previously reported that the timely removal of PARylation, controlled by PARG, contributes to efficient DNA damage repair, as depletion or inhibition of PARG has been shown to sensitize cells to different types of DNA-damaging agents.^56^^,^^57^^,^^58^^,^^59^ However, the cellular phenotypes were relatively mild, suggesting potential redundancies with other ADP-ribosylhydrolases.^56^^,^^59^ Indeed, we show here that the joint deficiency of TARG1 and PARG induces dramatic cell sensitivity even in the absence of exogenous DNA damage. The joint loss of TARG1 and PARG activity leads to a considerable enrichment of protein ADPr, indicating that TARG1 is required for efficient reversal of aspartate/glutamate-linked ADPr in cells. Of note, TARG1 loss was previously shown to sensitize cells to the bacterial toxin DarT, which ADP-ribosylates DNA bases.^60^^,^^61^^,^^62^

Similarly to the dual loss of TARG1 and PARG, the joint loss of ARH3, the serine-linked MAR hydrolase, and PARG has also been shown to induce a strong synergistic effect on ADPr levels, leading to cell toxicity.^25^ However, the mechanisms linking unregulated ADPr accumulation to cell toxicity are different for these two synthetic lethality interactions. The unregulated accumulation of serine-linked ADPr disrupts histone acetylation and induces transcriptional defects.^25^ In contrast, the unrestrained accumulation of aspartate/glutamate-linked ADPr described here induces high levels of replication stress and DSBs. Our observations are in line with the induction of replication stress underlying PARGi toxicity in a subset of ovarian cancer cells with pre-existing replication vulnerabilities.^31^^,^^63^ Our work additionally reveals that HPF1 deficiency further amplifies the toxic ADPr accumulation and replication stress phenotype induced by the joint loss of TARG1 and PARG. Knowing that HPF1 plays a role in the restriction of PAR chain length^12^^,^^64^^,^^65^ and that PARP1 activity is steered towards acidic sites upon the absence of HPF1,^11^^,^^12^^,^^36^ our results suggest that combining HPF1 deficiency with the joint loss of TARG1 and PARG further amplifies the accumulation of unregulated toxic ADPr.

The synthetic lethality interaction between TARG1 and PARG discovered here opens up promising therapeutic avenues. PARG downregulation, by leading to increased PARylation, has been reported to be a mechanism of PARPi resistance.^47^ Targeting TARG1 in these PARPi-resistant cancer cells with PARG downregulation could thus be a promising therapeutic strategy, highlighting the need to develop TARG1 inhibitors.

### Limitations of the study

While this study identifies TARG1 and PARG as important genome stability factors, it does not unravel the molecular mechanisms that enable the two enzymes to perform such roles. Future work is needed to uncover how the excessive accumulation of ADPr observed upon the joint loss of TARG1 and PARG induces replication stress in order to fully decipher the roles of the two enzymes in genomic stability.

## STAR★Methods

### Key resources table

**Table:** 

REAGENT or RESOURCE	SOURCE	IDENTIFIER
**Antibodies**		

anti-TARG1 (rabbit polyclonal)	Proteintech	Cat# 25249–1-AP; RRID:AB_2753118
anti-poly/mono ADPr (rabbit monoclonal)	Cell Signaling	Cat# 83732; RRID:AB_2749858
anti-H2AX (rabbit monoclonal)	Cell Signaling	Cat# 7631; RRID:AB_10860771
anti-CHK2 p-T68 (rabbit monoclonal)	Cell Signaling	Cat# 2197; RRID:AB_2080501
anti-CHK2 (rabbit polyclonal)	Cell Signaling	Cat# 2662; RRID:AB_2080793
anti-histone H3 (rabbit polyclonal)	Millipore	Cat# 07–690; RRID:AB_417398
anti-CHK1 p-S317 (rabbit polyclonal)	Cell Signaling	Cat# 2344; RRID:AB_331488
anti-PARG (rabbit monoclonal)	Cell Signaling	Cat# 66564 RRID:AB_2750890
anti-PARP1 (rabbit monoclonal)	Abcam	Cat# ab32138; RRID:AB_777101
anti-γH2AX (rabbit polyclonal)	Abcam	Cat# ab2893; RRID:AB_303388
anti-H3S10P (rabbit polyclonal)	Abcam	Cat# ab5176; RRID:AB_304763
anti-p53 p-S15 (mouse monoclonal)	Cell Signaling	Cat# 9286; RRID:AB_331741
anti-CHK1 (mouse monoclonal)	Cell Signaling	Cat# 2360; RRID:AB_2080320
anti-β-tubulin (rabbit polyclonal)	Abcam	Cat# ab6046; RRID:AB_2210370
anti-p53 (mouse monoclonal)	Santa Cruz	Cat# sc-126; RRID:AB_628082
anti-H3K9ac (rabbit monoclonal)	Cell Signaling	Cat# 9649; RRID:AB_823528
anti-laminA (rabbit polyclonal)	Abcam	Cat# ab290; RRID:AB_303395
anti-CtIP (rabbit polyclonal)	Abcam	Cat# ab70163; RRID:AB_1209429
anti-HPF1/C4orf27 (rabbit polyclonal)	NovusBio	Cat# NBP1-93973; RRID:AB_11005823
anti-GFP (rabbit polyclonal)	Abcam	Cat# ab290; RRID:AB_303395
anti-RPA32 p-S4/8 (rabbit polyclonal)	Bethyl	Cat# A300-245A; RRID:AB_210547
anti-RPA32 (rabbit polyclonal)	Bethyl	Cat# A300-244A; RRID:AB_185548
anti-KAP1 p-S824 (rabbit polyclonal)	Bethyl	Cat# A300-767A; RRID:AB_669740
anti-KAP1 (rabbit polyclonal)	Bethyl	Cat# A300-274A; RRID:AB_185559
anti-RPA32 p-T21 (rabbit polyclonal)	Abcam	Cat# ab61065; RRID:AB_946322
anti-RPA32 (mouse monoclonal)	Abcam	Cat# ab2175; RRID:AB_302873
anti-γH2AX (mouse monoclonal)	Millipore	Cat# 05–636; RRID:AB_309864
anti-γH2AX (rabbit monoclonal)	Cell Signaling	Cat# 9718; RRID:AB_2118009
Donkey polyclonal anti-mouse, Alexa Fluor 594-conjugated	Thermo Fisher Scientific	Cat# A-32787; RRID AB_2762830
Goat polyclonal anti-mouse, HRP-conjugated	Agilent	Cat# P0447; RRID:AB_2617137
Swine polyclonal anti-rabbit, HRP-conjugated	Agilent	Cat# P0399; RRID:AB_2617141
Goat polyclonal anti-rabbit, Alexa Fluor 488-conjugated	Thermo Fisher Scientific	Cat# A-11034; RRID:AB_2576217

**Chemicals, peptides, and recombinant proteins**		

PDD00017273	Sigma	Cat# SML1781
Olaparib	Cayman Chemical	Cat# 10621
Veliparib	Enzo Life Sciences	Cat# ALX-270-444M005
Methyl methanesulfonate (MMS)	Sigma	Cat# 129925
Hydrogen peroxide (H_2_O_2_)	Sigma	Cat# H1009
VE-821	Sigma	Cat# SML1415
Etoposide	Sigma	Cat# E1383
AZD5305	MedChemExpress	Cat# HY-132167
UPF 1069	Selleckchem	Cat# S8038
TransIT-LT1 Transfection Reagent	Mirus Bio	Cat# MIR 2300
cOmplete™, EDTA-free Protease Inhibitor Cocktail	Sigma	Cat# 11873580001
PhosSTOP	Sigma	Cat# 4906845001
Benzonase	Sigma	Cat# 1016970001
NuPAGE LDS sample buffer	Invitrogen	Cat# NP0007
TCEP	Sigma	Cat# 646547
NuPAGE Novex 4–12% Bis-Tris gel	Invitrogen	Cat# WG1402A
Hoechst 33342	Invitrogen	Cat# H3570
DAPI	Sigma	Cat# D9542
G-148 Sulfate solution	Gibco	Cat# 10131027

**Critical commercial assays**		

Lipofectamine RNAiMAX	Invitrogen	Cat# 3778075
Neon Transfection System	Invitrogen	Cat# MPK5000
Lipofectamine 3000	Invitrogen	Cat# L3000015
QuikChange Lightning Site-Directed Mutagenesis Kit	Agilent	Cat# 210519
Click-iT Plus EdU Alexa Fluor 647 Flow Cytometry Assay Kit	Invitrogen	Cat# C10419
LR Clonase II enzyme mix	Invitrogen	Cat# 11791020

**Experimental models: Cell lines**		

Human: U2OS cells	ATCC	Cat# HTB-96
Human: U2OS TARG1 KO cells	(Tromans-Coia et al., 2021)^62^	N/A
Human: U2OS TARG1 KO cells complemented with untagged TARG1 WT	This paper	N/A
Human: U2OS TARG1 KO cells complemented with untagged TARG1 K84A	This paper	N/A
Human: PEO1 cells	Gift from Scott H. Kaufmann (Mayo Clinic)	Cat# CVCL_2686
Human: PEO1 TARG1 KO cells	This paper	N/A
Human: U2OS DR-GFP cells	ATCC	Cat# CRL-3455

**Oligonucleotides**		

sgRNA targeting TARG1 exon 3 GGATTGTCGCATGGGCGCT	IDT	N/A
sgRNA targeting TARG1 intron 3 GGTAAACGTCTAAACTAG	IDT	N/A
Silencer™ Select TARG1.1 siRNA	Invitrogen	Cat# s48048
Silencer™ Select TARG1.2 siRNA	Invitrogen	Cat# s48049
Silencer™ Select CTiP siRNA	Invitrogen	Cat# s531736
Silencer™ Select Negative Control No. 1 siRNA	Invitrogen	Cat# 4390843
Silencer™ Select HPF1 siRNA	Invitrogen	Cat# s29883

**Recombinant DNA**		

pDONR221 (Gateway vector)	Invitrogen	Cat# 12536017
pDEST12.2 (Gateway vector)	Invitrogen	Cat# 11808-011
pcDNA3.1-mCherry	(Kleaveland et al., 2018)^66^	N/A
pCBASceI	Addgene	Cat# 26477
pDEST47-TARG1 WT (plasmid)	This paper	N/A
pDEST47-TARG1 K84A (plasmid)	This paper	N/A

**Software and algorithms**		

ImageJ	NIH	N/A
FlowJo	BD Biosciences	N/A
Prism 7	GraphPad	N/A
CellProfiler	(McQuin et al., 2018)^67^	N/A

### Resource availability

#### Lead contact

Further information and requests for resources and reagents should be directed to and will be fulfilled by the Lead Contact, Ivan Ahel (ivan.ahel@path.ox.ac.uk).

#### Materials availability

All research reagents generated by the authors will be made available on request from the lead contact.

### Experimental model and subject details

#### Cell culture

Human osteosarcoma U2OS (ATCC HTB-96) cells were acquired from ATCC and grown in DMEM (Sigma) supplemented with 10% FBS (Gibco) and penicillin-streptomycin (100 U/mL, Gibco). Human osteosarcoma U2OS DR-GFP (ATCC CRL-3455) were a gift from Maria Jasin (Memorial Sloan Kettering Cancer Center) and were cultured in DMEM (Sigma) supplemented with 10% FBS (Gibco) and penicillin-streptomycin (100 U/mL, Gibco). Human ovarian adenocarcinoma PEO1 (CVCL_2686) cells were a gift from Scott H. Kaufmann (Mayo Clinic) and were cultured in RPMI-1640 (Sigma) supplemented with 20% FBS (Gibco) and penicillin-streptomycin (100 U/mL, Gibco). U2OS TARG1-KO cells complemented with TARG1 WT or K84A were maintained in the presence of G418 (500 μg/mL, Gibco). All cell lines were cultured at 37°C with 5% CO_2_.

### Method details

#### Generation of cell lines

The U2OS TARG1 knock-out cell lines were generated as described previously.^62^ They were generated by CRISPR/Cas9 following the published protocol.^68^ The following gDNA sequences were targeted: CACCGAGGATTGTCGCATGGGCGCT; AAACAGCGCCCATGCGACAATCCTC. Annealed primers were cloned into pSpCas9(BB)-2A-GFP (PX458) and after sequencing verification, the plasmid was transfected into U2OS cells. 1–2 days post-transfection, single GFP-positive cells were sorted with a FACSAria II into 96-well plates. Monoclonal cell lines were tested for TARG1 deficiency by anti-TARG1 Western blot. pSpCas9(BB)-2A-GFP (PX458) was a gift from Feng Zhang (Addgene plasmid #48138).

The PEO1 TARG1-KO cells were generated by nucleofection of ribonucleoprotein (RNP) complexes, consisting of the Cas9 nuclease pre-loaded with sgRNA. The following sgRNA sequences were used: GGATTGTCGCATGGGCGCT (targets TARG1 exon 3); GGTAAACGTCTAAACTAG (targets TARG1 intron 3). For RNP formation, 22 pmol of hybridized crRNA/tracrRNA (equimolar amounts heated at 95°C for 5 min and cooled down slowly to RT) were mixed with 5 μg Cas9 HiFi protein (IDT) in a total volume of 2 μL IDT Duplex Buffer (IDT), resuspended 5 times and incubated at 37°C for 5 min. Resulting RNP mixes were added directly to cells prior to nucleofection. For nucleofection, cells were washed twice with PBS and nucleofected with 2 μL RNP mix in a total volume of 10 μL “buffer R″ using the Neon Transfection System (Invitrogen) with the following settings: 1400V - 15ms - 4 pulses. Nucleofected cells were seeded at low density and single-cell colonies were grown and propagated before being validated via DNA sequencing and Western blot.

For the complementation of U2OS TARG1-KO cells, plasmids expressing either pDEST47-GFP-tagged TARG1 WT or catalytically inactive K84A mutant were transfected using Lipofectamine 3000 (Invitrogen). 24 h later, media was replaced and supplemented with neomycin (600 μg/mL) and maintained for 2 weeks. Stable neomycin-resistant cells were collected by trypsinization whereupon transgene expression was assessed by Western blot analysis.

#### Cell proliferation assays

For colony formation assays, cells were plated at low densities in 6-well plates (1200 cells/well for U2OS WT cells; 1400 cells/well for U2OS TARG1-KO cells; 3000 cells/well for PEO1 WT cells; 15000 cells/well for PEO1 TARG1-KO cells) and grown in the indicated conditions for 11 days. For treatments with MMS (Sigma), Etoposide (Sigma) and H_2_O_2_ (Sigma), the cells were incubated with the indicated concentration of the drug in the medium for 1 h (MMS and Etoposide) or 20 min (H_2_O_2_) before being released into fresh medium and allowed to recover for 10 days. Cells were fixed and stained with 0.5% crystal violet in 25% methanol for 30 min, washed with water and air-dried. Quantification was performed using ImageJ/Fiji with the ColonyArea plugin.^69^ The surviving fraction at each dose was calculated after normalization to the plating efficiency of untreated samples. Each experiment was performed in triplicates.

#### Western blotting

When indicated, the cells were treated with 10 μM PARGi PDD00017273 (Sigma) or 0.1 μM Veliparib (Enzo Life Sciences) for 4 or 6 days. Cells were lysed with Triton X-100 lysis buffer (50 mM Tris-HCl pH 8.0, 100 mM NaCl, 1% Triton X-100) supplemented with 2.5 mM MgCl_2_, protease and phosphatase inhibitors (Roche), Olaparib (Cayman Chemical; 1 μM) and PARGi PDD00017273 (Sigma; 1 μM) at 4°C. The lysates were incubated with 0.05% Benzonase (Sigma) for 30 min at 4°C. Protein concentrations were analyzed by Bradford Protein Assay (BioRad). Proteins were boiled in 1x NuPAGE LDS sample buffer (Invitrogen) with TCEP (Sigma), resolved on NuPAGE Novex 4–12% Bis-Tris gels (Invitrogen), and transferred onto nitrocellulose membranes (BioRad) using Trans-Blot Turbo Transfer System (BioRad). The membranes were blocked in PBS buffer with 0.1% Tween 20 and 5% non-fat dried milk for 30 min at room temperature and incubated overnight with primary antibodies (1:1000, unless stated otherwise) at 4°C, followed by 1-h incubation with peroxidase-conjugated secondary anti-mouse (Agilent, P0447, 1:3000) or anti-rabbit (Agilent, P0399, 1:3000) antibody at room temperature.

Rabbit anti-TARG1 (25249–1-AP, 1:500) antibody was from Proteintech. Rabbit poly/mono-ADPr (83732), anti-H3K9ac (9649), anti-H2AX (7631), anti-CHK2 p-T68 (2197, 1:500), anti-CHK2 (2662), anti-CHK1 p-S317 (2344, 1:500), anti-PARG (66564, 1:500), anti-H3K9ac (9649), and mouse p53 p-S15 (9286, 1:500), CHK1 (2360) antibodies were from Cell Signaling. Mouse anti-p53 (sc-126) was from Santa Cruz. The antibody of rabbit anti-histone H3 (07–690, 1:5000) was from Millipore. Rabbit anti-PARP1 (ab32138; 1:2000), anti-γH2AX (ab2893), anti-H3S10P (ab5176), anti-β-Tubulin (ab6046), anti-Lamin-A (ab26300), anti-CtIP (ab70163) and anti-GFP (ab290, 1:3000) were from Abcam. Rabbit anti-RPA32 p-S4/8 (A300-245A,1:500), anti-RPA32 (A300-244A), anti-KAP1 p-S824 (A300-767A, 1:500), anti-KAP1 (A300-274A) were from Bethyl. Rabbit anti-HPF1/C4orf27 (NBP1-93973) antibody was from NovusBio. Blots were developed using ECL (Invitrogen) and analyzed by exposing them to films.

#### siRNA transfection

siRNA transfection was performed using Lipofectamine RNAiMAX (Invitrogen) and 20 nM siRNA for the indicated time according to the manufacturer’s instructions. Silencer Select Negative Control No. 1 siRNA, Silencer Select TARG1.1 (s48048), TARG1.2 (s48049), HPF1 (s29883) and CtIP (s531736) siRNAs were purchased from Ambion (Invitrogen).

#### Homologous recombination reporter assay

U2OS DR-GFP were seeded in 6 cm dishes and reverse transfected with siRNAs as described above. The following day, cells were transfected using TransIT-LT1 (Mirus) with 3.2 μg of a pCBASceI (gift from Maria Jasin). Parallel transfections with 0.8 μg of pcDNA3.1-mCherry^66^ were performed to assess the transfection efficiency. The cells were collected 2 days after transfection by resuspension of the cell pellet in 0.1 μg/mL DAPI solution in PBS. 20,000 DR-GFP cells were analyzed per condition on a Cytoflex LX (Beckman Coulter), using CytExpert version 2.3 (Beckman Coulter) for data collection. The GFP-positive population was determined by flow cytometry and normalized for transfection efficiency. Post-acquisition analysis was performed in FlowJo software (BD Biosciences).

#### Analysis of cell cycle and EdU incorporation

Cells were seeded in 6-well plates, treated as indicated and incubated with 10 μM EdU for 1 h at the end of treatment. Cells were harvested by trypsinization and labeled using the Click-iT Plus EdU Alexa Fluor 647 Flow Cytometry Assay Kit (Invitrogen) according to the manufacturer’s instructions. For the analysis of DNA damage levels, cells were then stained with γH2AX primary antibody (Cell Signaling, 9718S, 1:200) in 1% BSA in PBS for 30 min at room temperature, washed once and incubated for 30 min with Alexa Fluor 488-conjugated goat anti-rabbit secondary antibody (Thermo Fisher, A11034, 1:500) in 1% BSA in PBS. For DAPI staining, cell pellets were resuspended in 1 μg/mL DAPI solution (Sigma) in PBS and incubated protected from light for 5 min. Cells were washed in PBS and analyzed immediately after staining on Cytoflex LX (Beckman Coulter), using CytExpert version 2.3 (Beckman Coulter) for data collection (20,000 cells per sample were analyzed). Post-acquisition analysis was performed in FlowJo software (BD Biosciences).

#### Immunostaining and microscopy

For staining of ADPr, cells were seeded on glass coverslips and grown in the indicated conditions. Cells were washed with PBS, pre-extracted with 0.2% Triton X-100/PBS supplemented with 1 μM Olaparib and 1 μM PARGi PDD00017273 for 5 min and washed with PBS, then fixed with 4% paraformaldehyde (PFA, Sigma) for 15 min supplemented with 1 μM Olaparib and 1 μM PARGi PDD00017273, washed with PBS, permeabilized with 0.2% Triton X-100/PBS for 10 min and blocked with 10% FBS (GIBCO) in DMEM (Sigma) for 30 min. Incubation with primary rabbit antibody anti-poly/mono-ADPr (Cell Signaling, 83732, 1:330) was performed for 1 h at room temperature, followed by washing and 1-h incubation with Alexa Fluor 488-conjugated goat anti-rabbit secondary antibody (Thermo Fisher Scientific, A11034, 1:500). Coverslips were washed with PBS and counterstained with 0.1 μg/mL DAPI (4,6-diamidino-2-phenylindole, Sigma) in PBS for 10 min. After washing with PBS, coverslips were mounted onto glass slides with Mowiol 4–88 (Sigma). Images were acquired on Olympus Fluoview FV1200 confocal microscope using 40x/1.3 Oil UPlanSApo objectives under non-saturating conditions. Image quantification was performed using ImageJ/FIJI. Nuclei segmentation was performed using Huang thresholding and watershed. Identified nuclei objects were then used as a mask across all image channels and the pixel intensities for the GFP channel were measured.

For staining of RPA32 p-T21, RPA32 and γH2AX, cells were seeded on a 24-well glass bottom plate and grown in the indicated conditions. Cells were washed with PBS, and fixed with 20 mM Pipes-KOH, pH 6.8, 0.2% Triton X-100, 1 mM MgCl2, and 4% paraformaldehyde (PFA, Sigma) supplemented with 1 μM Olaparib and 1 μM PARGi PDD00017273 for 20 min, washed with PBS, permeabilized with 0.5% Triton X-100/PBS supplemented with 1 μM Olaparib and 1 μM PARGi PDD00017273 for 5 min and blocked with 5% BSA in PBS with 0.1% Tween 20 for 30 min. Incubation with primary rabbit anti-RPA32 pT21 antibody (Abcam, ab61065, 1:1000), mouse anti-RPA32 (Abcam, ab2175, 1:1000) and anti-γH2AX (Millipore, 05–636, 1:1000) antibodies was performed for 1 h at room temperature followed by washing and 1-h incubation with Alexa Fluor 488-conjugated goat anti-rabbit secondary antibody (Thermo Fisher Scientific, A11034, 1:500) or Alexa Fluor 594-conjugated donkey anti-mouse secondary antibody (Thermo Fisher Scientific, A32787, 1:500). Cells were washed with PBS and stained with 1 μg/mL Hoechst 33342 (Invitrogen) for 30 min. Images were acquired on the EVOS M7000 fluorescent microscope using 20X/0.75 UPlanSApo objectives under-non saturating conditions. Image quantification was performed using CellProfiler.^67^ Nuclei segmentation was performed using two-class Otsu thresholding. Identified nuclei objects were then used as a mask across all image channels. RPA32 p-T21, RPA32 and γH2AX foci were identified using three-class Otsu thresholding and nuclei respectively containing more than 5, 15 or 10 RPA32 p-T21, RPA32 and γH2AX foci were counted for each image.

### Quantification and statistical analysis

Prism 7 (GraphPad) was used for statistical analysis, where ^∗^p < 0.05, ^∗∗^p < 0.01, ^∗∗∗^p < 0.001, ^∗∗∗∗^p < 0.0001. Details of statistical analyses are described in the figure legends.

## Data Availability

• Data reported in this paper will be shared by the lead contact upon request. • This paper does not report original code. • Any additional information required to reanalyze the data reported in this paper is available from the lead contact upon request.

## Supplemental information

Document S1. Figures S1–S3

Document S2. Article plus supplemental information
