# Supplementary material for: The interplay of TARG1 and PARG protects against genomic instability
Source: Cell Rep. 2023 Sep 6;42(9):113113. doi: 10.1016/j.celrep.2023.113113 (PMC10933786; doi:10.1016/j.celrep.2023.113113)
Supplement: Document S1. Figures S1–S3 [file mmc1.pdf]

Cell Reports, Volume *42*

Supplemental information

The interplay of TARG1 and PARG protects against genomic instability

Joséphine Groslambert, Evgeniia Prokhorova, Anne R. Wondisford, Callum Tromans-Coia, Celeste Giansanti, Jennifer Jansen, Gyula Timinszky, Matthias Dobbelstein, Dragana Ahel, Roderick J. O'Sullivan, and Ivan Ahel

Figure S1. The loss of TARG1 sensitizes cells to ATR inhibition and induces homologous recombination defects, related to Figure 1

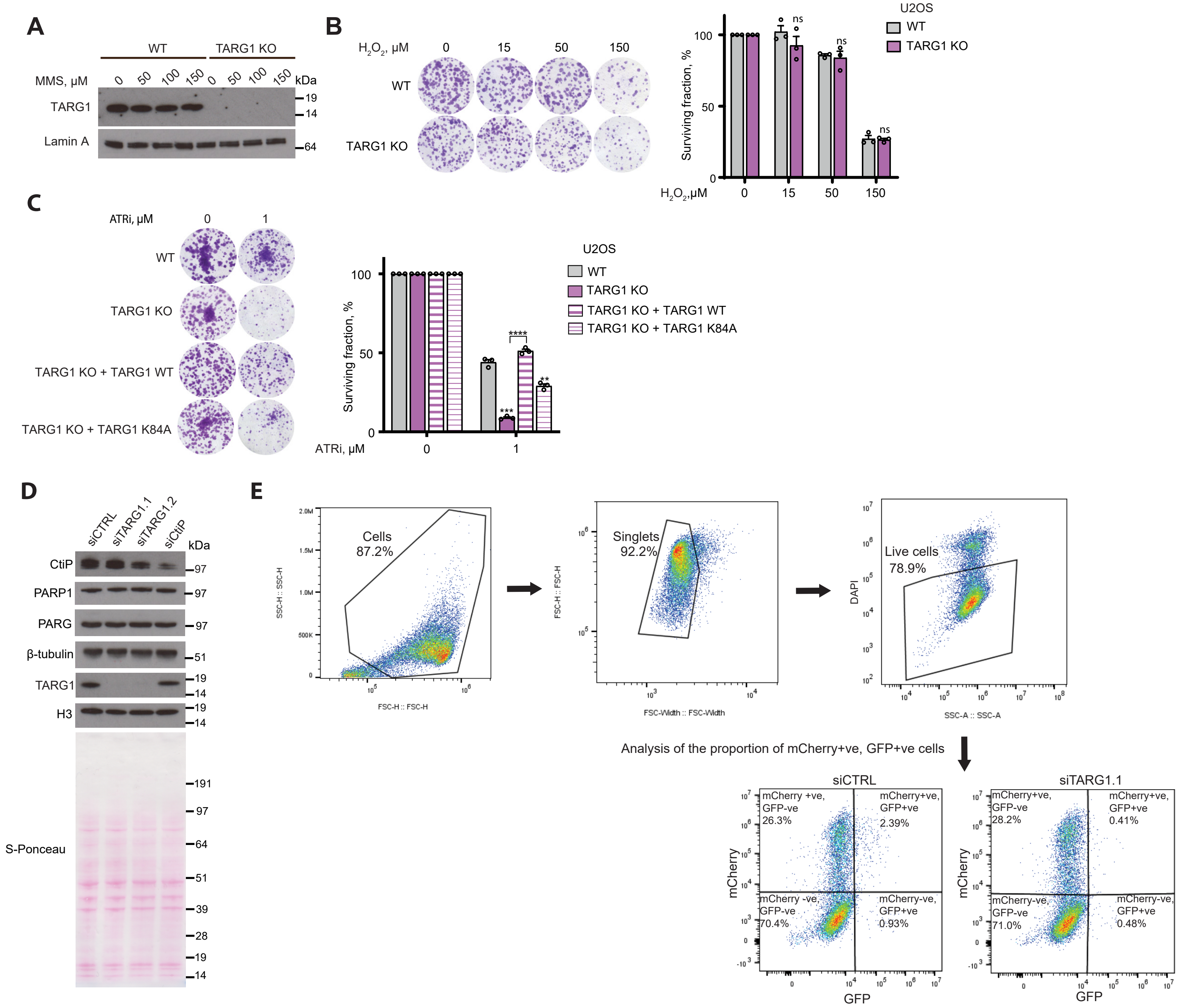

Figure S2. TARG1 deficiency is synthetically lethal with PARG suppression, related to Figure 2

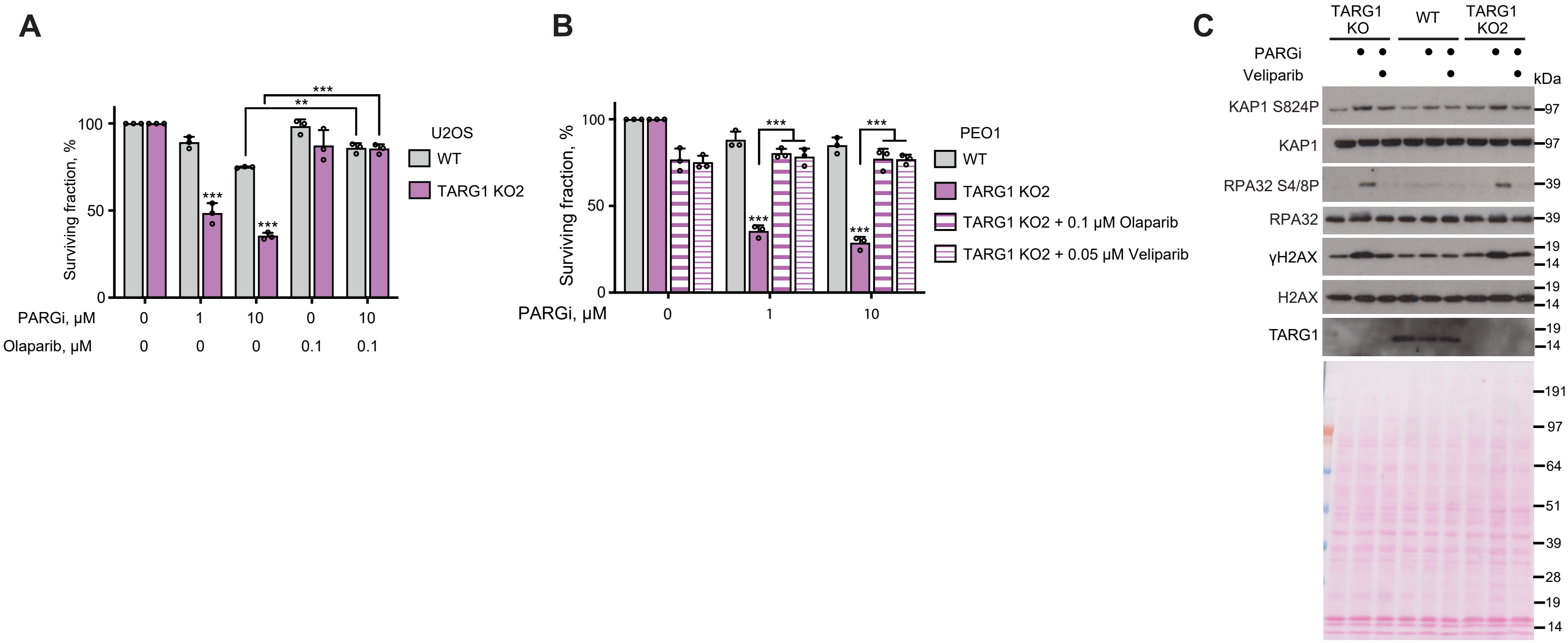

Figure S3. The joint loss of TARG1 and PARG activity induces replication stress, related to Figure 3

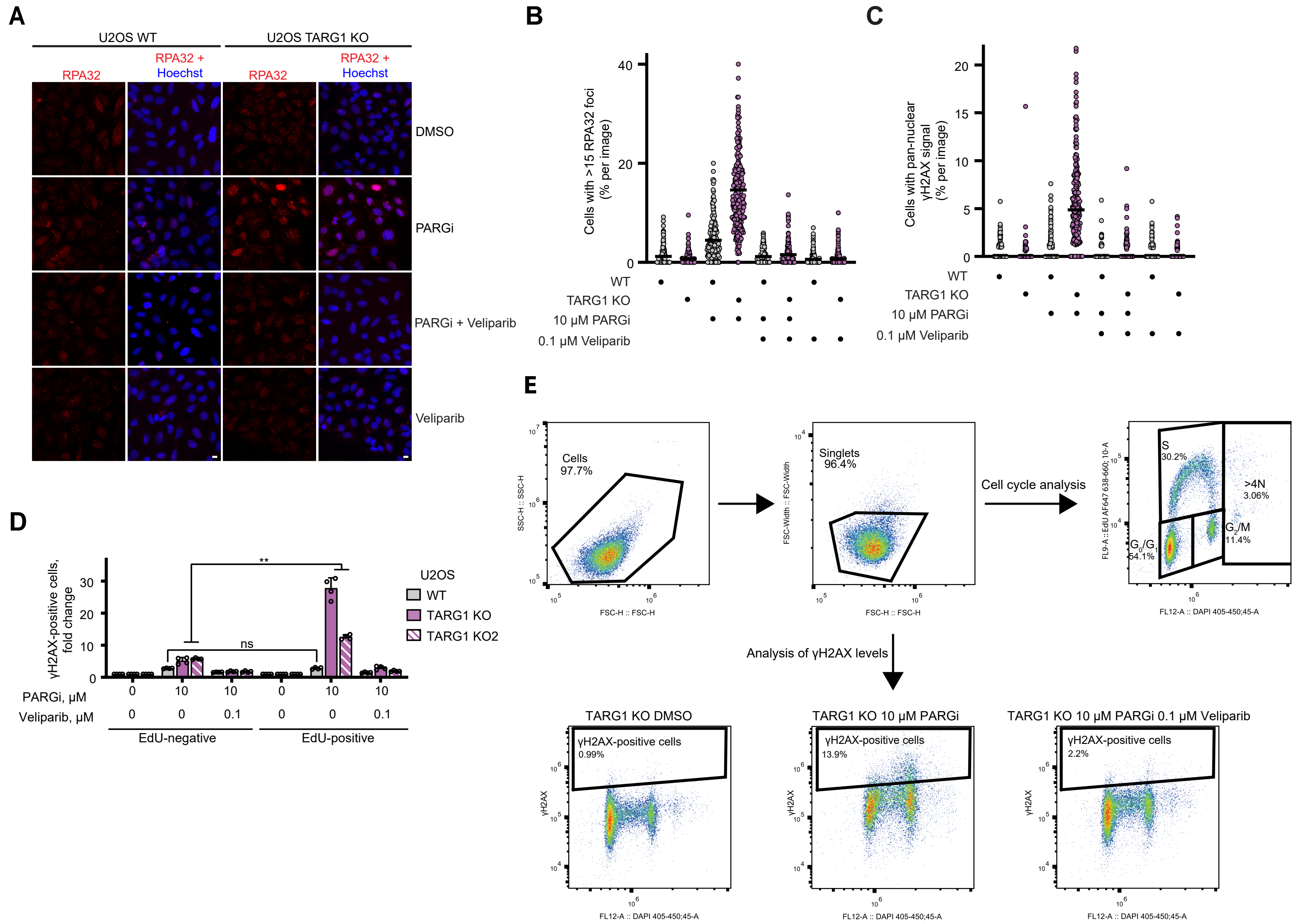

Supplemental Figure Legends

Figure S1. The loss of TARG1 sensitizes cells to ATR inhibition and induces homologous recombination, related to Figure 1

(A) U2OS cells were treated with DMSO or MMS at the indicated concentration for 1 h. TARG1 levels were analysed using western blotting.

(B and C) Representative images (left) and quantification (right) of colony formation assay with U2OS WT and TARG1-KO cells (B and C), and TARG1-KO cells complemented with TARG1 WT or catalytically inactive K84A mutant (C) treated with DMSO or as indicated.

(D) U2OS DR-GFP cells were transfected with siCTRL, two different siTARG1 or siCtIP for 72 h prior to TARG1 levels analysis by western blotting.

(E) Flow cytometry gating strategy for analyzing the proportion of GFP positive cells amongst the mCherry positive population in Figure 1F.

Data are shown as mean ± SD, ns, not significant, $**p < 0.01$, $***p < 0.001$ and $****p < 0.0001$ (two-tailed Student's t test).

Figure S2. TARG1 deficiency is synthetically lethal with PARG suppression, related to Figure 2

(A and B) Quantification of colony formation assay with U2OS WT and an independent TARG1-KO clone (A) and PEO1 WT and an independent TARG1-KO clone (B) treated with DMSO or as indicated.

(C) The levels of TARG1 and DNA damage markers of PEO1 WT cells and two PEO1 TARG1-KO clones were analysed by western blotting.

Figure S3. The joint loss of TARG1 and PARG activity induces replication stress, related to Figure 3

(A) Representative images of RPA32 staining in U2OS cells treated with DMSO, 10 µM PARGi, 10 µM PARGi and 0.1 µM Veliparib or 0.1 µM Veliparib for 4 days. Scale bars, 10 µm. A representative image from n = 2 is shown.

(B) Quantification of (A). Each point represents the percentage of cells with >15 RPA32 foci per image. ~250 images and a total of ~20000 cells were analysed per condition.

(C) Quantification of (Figure 3D). Each point represents the percentage of cells with pan-nuclear γH2AX signal per image. 250 images and a total of ~20000 cells were analysed per condition.

(D) Flow cytometry quantification of γH2AX-positive cells amongst the EdU-negative and -positive cell population after 5 days of exposure to DMSO or indicated treatment. After drug treatment, cells were incubated with 10 µM EdU for 1 h prior to being stained

with γH2AX primary antibody and analyzed by flow-cytometry. Data are shown as mean ± SEM of four independent experiments. **$p < 0.01$ (two-tailed Student's t-test).

(E) Flow cytometry gating strategy for analyzing γH2AX levels in Figure 3G and cell cycle distribution in Figure 3I.
